# Observation of Magnetic Pseudogap Behavior in Phosphorus‐Doped Silicon

**DOI:** 10.1002/advs.202502789

**Published:** 2025-07-25

**Authors:** Suheon Lee, Sangeun Cho, Yongcheol Jo, Wonjun Lee, Jae Min Kim, Hong Gu Lee, Yugo Oshima, Taku Matsushita, Hiroki Ikegami, Jonas A. Krieger, Christoper Baines, Thomas J. Hicken, Hubertus Luetkens, Eundeok Mun, Jungseek Hwang, Hyunsik Im, Kwang‐Yong Choi

**Affiliations:** ^1^ Center for Artificial Low Dimensional Electronic Systems Institute for Basic Science Pohang 37673 Republic of Korea; ^2^ Division of System Semiconductor Dongguk University Seoul 04620 Republic of Korea; ^3^ Research Center for Dielectric and Advanced Matter Physics Pusan National University Busan 46241 Republic of Korea; ^4^ Department of Physics Sungkyunkwan University Suwon 16419 Republic of Korea; ^5^ Nuclear Spectroscopy Laboratory RIKEN Pioneering Research Institute Hirosawa 2‐1, Wako Saitama 351‐0198 Japan; ^6^ Department of Physics Nagoya University Chikusa‐ku Nagoya 464‐8602 Japan; ^7^ Beijing National Laboratory for Condensed Matter Physics Institute of Physics Chinese Academy of Sciences Beijing 100190 China; ^8^ PSI Center for Neutron and muon Sciences CNM Villigen 5232 Switzerland; ^9^ Department of Physics Simon Fraser University Burnaby British Columbia V5A 1S6 Canada

**Keywords:** inhomogeneous Kondo clouds, Kondo condensate, phosphorus‐doped silicon, pseudogap

## Abstract

The recent discovery of a Kondo condensate in phosphorus‐doped silicon (Si:P) presents its significant potential for achieving novel many‐body quantum states. Si:P exhibits Kondo condensation, characterized by an energy gap in the electronic density of states, while the precise nature of its magnetic state has yet to be determined. Here, we utilize electron and muon spin resonance (ESR and *µ*SR) techniques, optical spectroscopy, and specific heat measurements to unravel the magnetic ground state and spin dynamics of Si:P. Both optical and ESR spectroscopy reveal the onset of spin correlations below 150 K. Furthermore, the muon spin relaxation rate exhibits a power‐law increase, λ_ZF_∼*T* ^−0.26(5)^, below *T*_KC_ ≈ 0.2 K, indicating the emergence of critical spin fluctuations within the Kondo condensate state. Strikingly, the concomitant occurrence of a Bardeen‐Cooper‐Schrieffer‐like charge gap and power‐law magnetic fluctuations closely parallels the pseudogap phases observed in doped Mott insulators. These findings evince that the critical spin fluctuations of the Kondo condensate state act as a driving force for pseudogap formation within inhomogeneous Kondo clouds.

## Graphical abstract

Phosphorus‐doped silicon presents a promising platform for quantum computing and hosts a Kondo condensate state featuring a pseudogap. Thermodynamic and magnetic resonance measurements reveal that this pseudogap behavior involves critical spin fluctuations, suggesting that overlapping Kondo clouds form a correlated coherent magnetic ground state, closely resembling the pseudogap phase of doped Mott insulators.

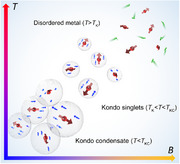

## 1 Introduction

Silicon, the cornerstone of modern semiconductor technology, is now being investigated as a scalable platform for quantum computing, thanks to its well‐established fabrication infrastructure.^[^ ^1^ ^]^ In addition to its technological applications, doped silicon has served as a key platform for exploring quantum many‐body phenomena occurring at the transition between metallic and insulating states.^[^ ^2^, ^3^, ^4^, ^5^, ^6^, ^7^ ^]^ This insulator‐metal transition is particularly captivating, as it involves the formation of a Coulomb glass in the insulating state and the emergence of superconductivity in the degenerately doped metallic state of boron‐doped silicon. Singularly, the Kondo effect appears just above the metallic threshold, offering a unique window into the interplay of spatial inhomogeneity, magnetism, and electron correlations.

**Figure 1:**
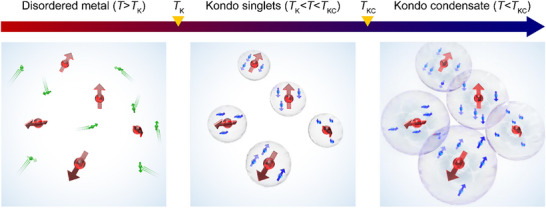
(Left) At high temperatures *T* > *T*_K_, conduction electrons (green spheres and arrows) move nearly freely, developing weak antiferromagnetic correlations near magnetic impurities (red spheres and arrows). (Middle) In the intermediate temperature range, *T*_KC_ < *T* < *T*_K_, Kondo singlets (transparent spheres) are formed by the Kondo coupling between the impurity spins and conduction electron spins (blue spheres and arrows). (Right) At low temperatures *T* < *T*_KC_, Kondo condensate emerges from the condensation of overlapping Kondo singlets.

In systems with low concentrations of magnetic impurities, Kondo‐screened quasibound states form isolated singlet (**Figure** **1**).^[^ ^8^ ^]^ When dense magnetic impurities are periodically arranged, they create a Kondo lattice, or a local moment lattice, characterized by coherent scattering below the Kondo temperature. This coherent state results in two key ramifications: a pronounced reduction in resistivity and the opening of a hybridization gap between the Kondo lattice and conduction bands, enabling Kondo insulators and heavy fermion states. However, the intermediate regime‐where randomly distributed Kondo clouds overlap and interact‐remains poorly understood. Thus, bridging the behaviors of isolated Kondo clouds and the coherent Kondo lattice poses a pressing challenge in the field.

In the intermediate densities of magnetic impurities, a long‐sought correlated ground state termed the Kondo condensate was recently discovered in heavily phosphorus‐doped bulk silicon (Si:P).^[^ ^6^, ^7^ ^]^ Phosphorus doping in silicon introduces impurity states near the conduction band edge. At high doping concentrations of *n* ∼ (2–5) × 10^19^ cm^−3^, these impurity states begin to overlap and form impurity bands that eventually merge with the conduction band, effectively shifting the Fermi level into the conduction band.^[^ ^9^, ^10^ ^]^ This results in a metallic state where localized magnetic moments can emerge due to incomplete screening and electron correlation effects. In the vicinity of the metal‐insulator transition, many‐body interactions, disorder, and carrier localization play a crucial role in shaping the electronic and magnetic properties of Si:P.

In the heavily doped Si:P, differential resistance *R*_d_ reveals a transition from Fermi liquid to a Kondo‐singlet‐like state at *T*_K,d_ ∼ 1 K, eventually forming a correlated yet disordered Kondo lattice.^[^ ^6^ ^]^ This exotic state arises from the condensation of moderately overlapping Kondo clouds, where itinerant electrons are partially bound to magnetic impurities (Figure 1). In the Kondo condensate, tunneling density‐of‐states spectroscopy shows an energy gap in charge excitations, akin to the gap in the density of states observed in Bardeen‐Cooper‐Schrieffer (BCS) superconductors.^[^ ^4^ ^]^ Nonetheless, nothing is known about the magnetic nature of the alleged macroscopically coherent state, although the Kondo effect competes with Ruderman–Kittel–Kasuya–Yoshida (RKKY) magnetic interactions.

To address this, we integrate resonance techniques, optical spectroscopy, and specific heat measurements, providing evidence that the pseudogap in the Kondo condensate state originates from critical spin fluctuations of overlapping Kondo clouds. The simultaneous emergence of a partial charge gap and critical magnetic correlations bears parallels to the pseudogap state found in doped Mott insulators.^[^ ^11^ ^]^ These similarities underscore the intricate interplay between nonlocal Kondo condensation and RKKY‐mediated spin fluctuations in stabilizing the enigmatic pseudogap phases in strongly interacting, inhomogeneous Kondo‐singlet clouds.

## 2 Results

### 2.1 Crossover From Disordered Metal to Kondo Singlet

We first examine the temperature evolution from the paramagnetic Fermi liquid phase to the weakly magnetic metallic phase through optical spectroscopy and electron spin resonance (ESR). These complementary techniques illuminate the electronic and magnetic characteristics that drive the formation of the Kondo condensate.

**Figure 2:**
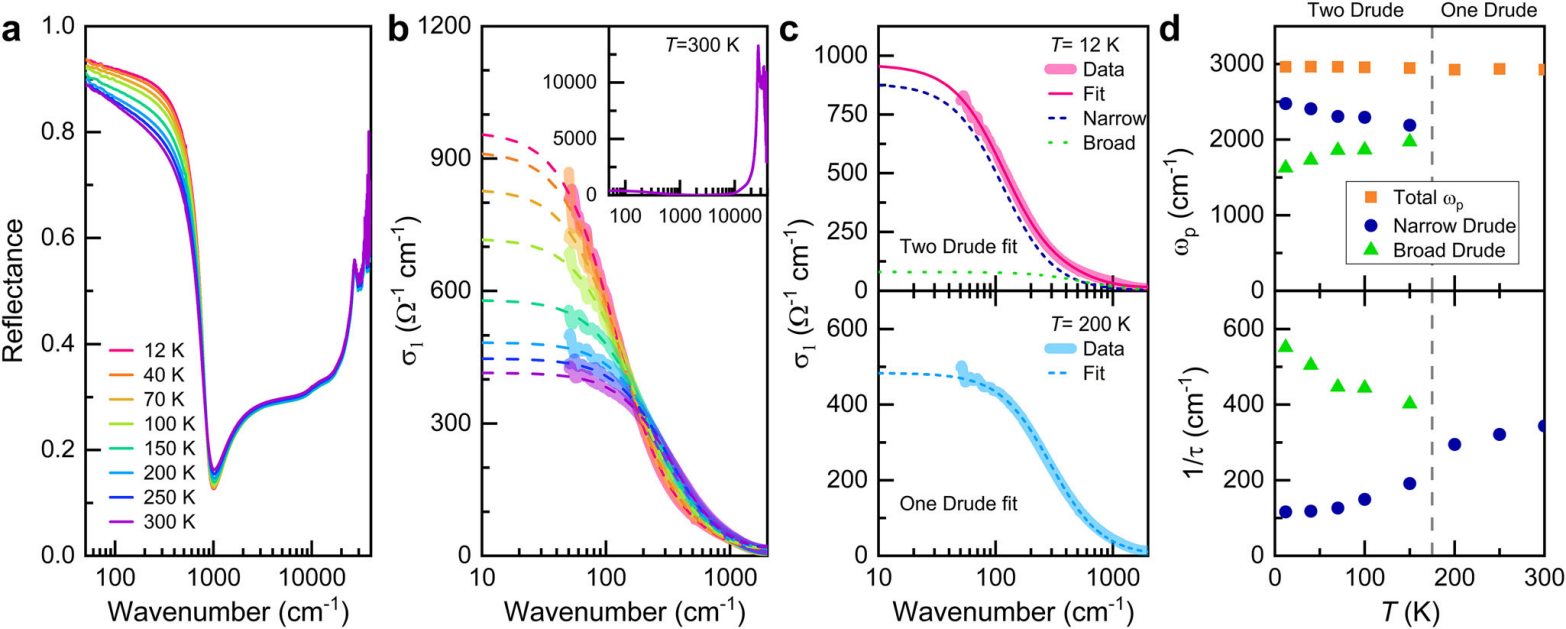
a) Reflectance spectra of Si:P at various temperatures. b) Optical conductivity of Si:P at different temperatures obtained using Kramers‐Kronig analysis. The thick light curves and dashed lines represent the data and the fittings. The inset shows the optical conductivity of Si:P in a wide wavenumber range up to 40 000 cm^−1^ at *T* = 300 K. c) Drude analysis of the optical conductivity at two representative temperatures. d) Temperature dependence of the plasma frequency and impurity scattering rate extracted from the Drude analysis.

**Figure** **2**a presents the reflectance spectra of the Si:P samples measured over a wide spectral range from the far infrared to ultraviolet at various temperatures from 12 to 300 K. The reflectance spectra display metallic behavior, with an enhancement in low‐frequency reflectance as the temperature is lowered. A plasma edge near 800 cm^−1^, related to a charge carrier density, indicates a small charge carrier density in the Si:P sample. Shown in Figure 2b are the *T*‐dependent optical conductivity spectra derived from the measured reflectance spectra using Kramers‐Kronig analysis.^[^ ^12^ ^]^ The optical conductivity exhibits a Drude‐like peak at zero frequency, indicating the presence of charge carriers. To analyze this behavior, we fitted the low‐frequency optical conductivity to the Drude model,^[^ ^12^ ^]^ with the fit results shown in Figure 2c. The inset in Figure 2b shows the conductivity in a wide range up to 40 000 cm^−1^, where a strong interband transition caused by the energy gap dominates at high frequencies.

Notably, a single Drude model could not adequately describe the data below 200 K, as exemplified by two representative data with Drude‐model fits at *T* = 12 K and 200 K in Figure 2c. Therefore, we employed two‐mode (narrow and broad) Drude model for the fits below 200 K, yielding two parameters: the plasma frequency (*ω*_p_) and the impurity scattering rate (1/*τ*).^[^ ^12^ ^]^ Figure 2d plots the *T*‐dependent fitting parameters. The scattering rate (1/*τ*_n_) of the narrow Drude mode exhibits the typical temperature behavior of a simple metal: as the temperature decreases, the scattering rate decreases. However, the scattering rate of (1/*τ*_b_) of the broad Drude mode shows an opposite *T* dependence, alluding to a spectrum of itinerant carrier dynamics, which arises from intriguing interactions between disorder, electron‐electron correlation, and magnetic fluctuations. In this context, the narrow Drude component corresponds to carriers with relatively low scattering rates, typically associated with coherent quasiparticles. In contrast, the broad Drude component captures more incoherent carriers, which are strongly scattered likely due to interactions with fluctuating local moments or disorder‐related effects. Additionally, as the temperature rises, the plasma frequency (*ω*_p,n_) of the narrow Drude mode slightly decreases, while that of the broad Drude mode increases by the same amount, maintaining the total plasma frequency (*ω*_p,t_) constant. The total charge carrier density is roughly estimated as *n* ∼ 9.4 × 10^19^ cm^−3^ from the total plasma frequency using the relation *ω*_p,t_^2^ = 4π*ne* ^2^/*m*_e_, where *e* is the unit charge and *m*_e_ is the electron mass.

**Figure 3:**
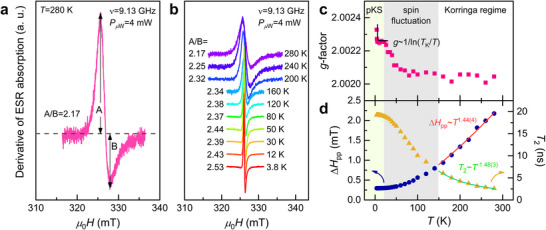
a) Representative Dysonian ESR lineshape of Si:P measured at *T* = 280 K with *P* = 4 mW. **b** ESR spectra recorded at various temperatures. c) *g*‐factor as a function of temperature. The solid curve represents the fit of the data using the single‐ion Kondo model. d) Temperature dependence of the peak‐to‐peak ESR linewidth Δ*H*_pp_ (left axis) and the spin‐spin relaxation time *T*_2_ (right axis). The solid lines are guides for the power‐law dependence of Δ*H*_pp_ and *T*_2_. The light green and gray shaded areas mark the precursor Kondo‐singlet (pKS) regime below 20 K and the spin‐fluctuation regime for 20 < *T* < 150 K, respectively.

To shed light on the exchange coupling between localized and itinerant moments, we further conducted ESR experiments on Si:P. As shown in **Figure** **3**a, the ESR signal at *T* = 280 K exhibits a well‐defined Dysonian lineshape in the diffusionless regime (*A/B* = 2.17), as expected for local moments in a metallic environment (see **Methods** for details).^[^ ^13^, ^14^ ^]^ With decreasing temperature, the ESR spectra become narrower and slightly more diffusive (*A*/*B* = 2.17→ 2.53), as illustrated in Figure 3b. The microwave power dependence of the ESR spectra shows a gradual broadening as the microwave power increases, followed by saturation of the ESR intensity above *P_µ_*_W_ = 100 mW (Figure S1a–c, Supporting Information). The diffusionless character of the ESR lineshape remains robust against temperature (*α*_avg_ ∼ 0.45 for *T* = 3.8–280 K) and microwave power (0.40 < *α* < 0.48 for *P_µ_*_W_ = 0.1–200 mW).

For quantitative analysis, we fit the ESR spectra using the admixture of the absorption and dispersion of Lorentzian lineshapes (**Methods**). The lineshape parameters, the *g*‐factor, and the peak‐to‐peak linewidth Δ*H*_pp_(*T*) are plotted in Figure 3c,d and Figure S1b–e (Supporting Information). In the simple paramagnetic regime for *T* > 150 K, the effective *g*‐factor (*g*_eff_ ∼ 2.0020) is temperature‐independent (Figure 3c). Upon further cooling, the *g*‐factor experiences a moderate increase through 60 K and then saturates to a constant value down to 20 K, and finally shows a sudden rise below 6 K. We recall that the *g*‐factor shift is given by $\Delta g\propto N\left(E_{F}\right)J_{\mathrm{ce}-S}\left(q=0\right)$, where *J*_ce‐S_ is the exchange integral between the conduction electrons and the local moments, and *N*(*E*_F_) is the electronic density of states at the Fermi energy *E*_F._^[^ ^15^ ^]^ In this regard, the observed *g*‐factor shift points to a crossover to the spin‐fluctuation regime. We note that the spin fluctuation regime (20 < *T* < 150 K) in ESR data is associated with the appearance of an additional Drude component below ∼175 K in the optical spectroscopy results.

Remarkably, the steep increment observed at *T* < 6 K is reminiscent of a single‐ion Kondo scheme in diluted alloys and heavy‐fermion systems.^[^ ^16^, ^17^ ^]^ Within this scenario, the *T* dependence of the *g*‐factor is described by *g*(*T*) = *g*_0_ + *C*/ln(*T*_K,ESR_/*T*), where *g*_0_ is the offset value and *T*_K,ESR_ is the characteristic spin‐fluctuation temperature. The best fit is obtained with *g*_0_ = 2.0022(6) and *T*_K,ESR_ = 5.5(6) K. Using this value of *T*_K,ESR_, we estimate the peak‐to‐peak ESR linewidth as $\Delta H_{\mathrm{pp}}=k_{B}T_{K,\mathrm{ESR}}/\sqrt{3}\mu_{B}=4.72\;\mathrm{mT}$, slightly larger than the observed linewidth. We emphasize that the evaluated *T*_K,ESR_ is linked to the onset of the logarithmic temperature dependence in the differential resistance *R*_d_.^[^ ^6^ ^]^

Figure 3d exhibits the *T* dependence of Δ*H*_pp_(*T*), which measures the spin‐spin relaxation time (*T*_2_). With decreasing temperature, Δ*H*_pp_(*T*) initially decreases and then shows a continuous change in its slope through 150 K, eventually becoming temperature‐independent below 20 K. A power‐law fit to the high‐temperature regime yields the power‐law behavior *T* ^1.44(4)^ (see Figure S8, Supporting Information for details). Notably, this power‐law behavior closely aligns with recent ESR measurements on Si with a doping concentration of 7 × 10^19^ cm^−3^ above 100 K, which report a *T* ^3/2^ dependence of Δ*H*_pp_.^[^ ^18^ ^]^ This observed *T* ^3/2^ temperature dependence of Δ*H*_pp_ in heavily doped Si is interpreted within the framework that spin relaxation is primarily governed by scattering of conduction electrons off localized magnetic moments or donor‐induced disorder. In contrast, the Δ*H*_pp_(*T*)∼*T* ^1.44(4)^ dependence deviates significantly from the *T* ^3^ behavior predicted by the conventional Elliott‐Yafet spin‐orbit scattering mechanism in *n*‐type silicon.^[^ ^19^, ^20^, ^21^, ^22^ ^]^

Given the presence of inhomogeneous Kondo clouds in Si:P, a Korringa‐type relaxation mechanism may contribute to the behavior of Δ*H*_pp_(*T*), as observed in other Kondo systems.^[^ ^23^, ^24^, ^25^ ^]^ However, the power‐law increase for *T* > 150 K is significantly steeper than the linear *T* dependence expected for the Korringa relation, $\Delta H_{\mathrm{pp}}\propto \left\langle J_{\mathrm{ce}-S}^{2}\left(q\right)\right\rangle N^{2}\left(E_{F}\right)T$. Under these conditions, it is unclear whether the ESR signal primarily originates from localized spins being relaxed by conduction electrons, or from conduction electrons being scattered by localized spins. It is likely that several competing processes contribute to the linewidth broadening, including Korringa‐type relaxation, conventional spin‐orbit‐coupled electron scattering involving impurities and phonons, as well as spin fluctuations.

Taken together, metallic Si:P exhibits a gradual crossover from the paramagnetic regime to the spin‐fluctuation regime over a wide temperature range of *T* = 20–150 K, accompanied by the shift in *g*(*T*) and the slope change of Δ*H*_pp_(*T*) and *T*_2_(*T*)^[^ ^26^ ^]^ as well as the appearance of the two Drude modes. The *T*‐independent behaviors of *g*(*T*), Δ*H*_pp_(*T*), and *T*_2_(*T*) suggest that Kondo‐singlet fluctuations start to develop below 20 K. Eventually, Kondo singlets are formed below 6 K, resulting in a large Pauli‐like susceptibility in the applied field and the subsequent upturn in *g*(*T*).^[^ ^27^, ^28^ ^]^

### 2.2 Critical Spin Fluctuations in Kondo Condensate

**Figure 4:**
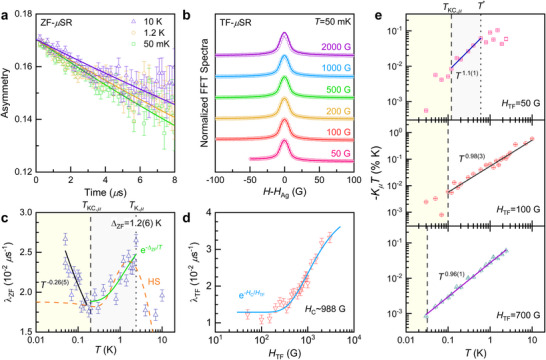
a) Representative ZF‐*µ*SR spectra at selected temperatures. The solid lines denote the fits to the data (**Methods**). b) Normalized FFT amplitudes of TF‐*µ*SR spectra at 50 mK. The solid curves represent the fitting lines (**Methods**). c) Zero‐field muon spin relaxation rate vs. temperature in a semilog scale. The blue and magenta solid lines indicate the power‐law dependence λ_ZF_∼*T* ^−0.26(5)^ and the activation behavior λ_ZF_ ∼ [1 + exp(‐Δ_ZF_/*T*)] with Δ_ZF_ = 1.2(6) K, respectively. The orange dashed curve denotes the fits to the Hebel‐Slichter peak. d) TF dependence of the TF muon spin relaxation rate in a semilog scale. The solid curve is the field‐dependent activation function λ_TF_ ∼ [1+exp(‐*H*_C_/*H*_TF_)] with the characteristic field *H*_C_ ∼ 988 G. e) Temperature dependence of the muon Knight shift multiplied by temperature ‐*K_µ_T*. The solid curves represent the power‐law behavior ‐*K_µ_T* ∼ *T^ξ^*. The dotted and dashed vertical lines in (c) and (e) indicate the characteristic temperature *T* ^*^ and *T*_KC_, respectively. The shaded regions in (c) and (e) represent the correlated Kondo singlet phase for *T*_KC_ < *T* < *T* ^*^ and the Kondo condensate state below *T*_KC_.

To explore the thermal and field evolution of spin correlations across the Kondo condensate transition, we performed muon spin relaxation/rotation (*µ*SR) experiments of Si:P in zero and transverse fields (TFs). As shown in **Figure** **4**a, the zero‐field (ZF) *µ*SR spectra show a slow exponential relaxation persisting down to 50 mK, indicative of dominant dynamic magnetism (**Methods**). The application of a 50 G longitudinal field leads to a decoupling of the longitudinal relaxation, suggesting a small energy scale of the exchange coupling (see Figure S2, Supporting Information for details). Figure 4b shows the normalized fast Fourier transformed (FFT) amplitudes of the TF‐*µ*SR spectra at *T* = 50 mK. The TF‐*µ*SR spectra display an exponential lineshape for all applied fields. With increasing field, the TF‐*µ*SR spectra steadily broaden without developing additional peaks. This, together with the exponentially relaxing ZF‐*µ*SR signals without coherent muon oscillation, gives no indication of weak magnetic order in the Kondo condensation (Figure 4a; Figure S3a, Supporting Information).

In Figure 4c, we plot the muon spin relaxation rate as a function of temperature in zero field. Upon cooling below 3 K, λ_ZF_(*T*) exhibits a λ‐like peak at *T*_K,_ *_µ_* = 2.4 K. This anomaly is reminiscent of the Hebel‐Slichter coherence peak in conventional BCS superconductors.^[^ ^29^, ^30^ ^]^ However, the Hebel‐Slichter model provides a poor description of the anomaly at *T*_K,_ *_µ_* (dashed line in Figure 4c). Alternatively, we fit the decrease of λ_ZF_(*T*) in terms of a thermally activated process λ_ZF_(*T*)∝1 + exp (− Δ_ZF_/*T*) with Δ_ZF_ = 1.2(6) K (green line). The characteristic temperature *T*_K,_ *_µ_* aligns closely with the onset temperature of the logarithmic dependence *R*_d_ ∼ ‐log(*T*).^[^ ^6^ ^]^ Thus, the thermal activation behavior of λ_ZF_(*T*) signals the formation of a Kondo‐ singlet‐like state below *T*_K,_ *_µ_*. Given the small reduction in λ_ZF_(*T*) over the temperature range of 0.2–2 K, however, the extracted parameter Δ_ZF_ should not be interpreted as a true gap. Rather, it is a phenomenological measure of the suppression of spin relaxation, associated with the emergence of spin‐singlet correlations. The most salient observation is the power‐law increase of λ_ZF_(*T*) ∼ *T* ^−0.26(5)^ below *T*_KC,_ *_µ_* ≈ 0.2 K, suggesting a slowdown of magnetic fluctuations. We emphasize that *T*_KC,_ *_µ_*, inferred from the ZF‐*µ*SR data, coincides with the Kondo condensation temperature *T*_KC,d_ ∼ 80–150 mK deduced from tunneling conductance (Figure S5, Supporting Information and Ref. [3]). This correspondence demonstrates that the transition from a Kondo‐singlet‐like state to Kondo condensation is accompanied by a drastic change of spin dynamics.

Noteworthy is that the thermal evolution of λ_ZF_ in Si:P differs markedly from that observed in heavy‐fermion Kondo systems without long‐range magnetic order,^[^ ^31^, ^32^, ^33^ ^]^ in which the muon spin relaxation rate shows persistent spin dynamics. In contrast, the unconventional temperature dependence of λ_ZF_(*T*) in Si:P (Figure 4c) conveys characteristics similar to the pseudogap phase in hole‐doped cuprates.^[^ ^34^, ^35^ ^]^ Specifically, in YBa_2_Cu_3_O_y_, the dynamic muon spin relaxation rate exhibits a maximum followed by a low‐*T* increase with decreasing temperature. The initial peak is ascribed to pseudogap formation, while the subsequent power‐law‐like increase stems from the critical slowing down of magnetic fluctuations.^[^ ^35^ ^]^ It is striking that the Kondo condensation in metallic Si:P displays the same pseudogap phenomenology observed in hole‐doped cuprates, involving intertwined fluctuations of charges and spins. However, unlike in cuprates, the pseudogap state associated with the Kondo condensation is not a precursor to symmetry‐broken states such as superconductivity or superfluidity.

We now inspect the TF dependence of the muon spin relaxation rate, as shown in Figure 4d. At low fields, λ_TF_(*H*_TF_) is nearly constant at ≈0.31 *µ*s^−1^ up to 200 G. As the field increases to 3 kG, λ_TF_(*H*_TF_) exhibits an *S*‐shaped increase. The field‐dependent λ_TF_(*H*_TF_) is well described by the activation function λ_TF_(*H*_TF_)∝1 + exp (− *H_C_*/*H*_TF_) with a characteristic field *H*_C_ ∼ 988 G. We stress that this exponential growth of λ_TF_(*H*_TF_) cannot be explained by inhomogeneous broadening or diamagnetic contributions (Figure S6, Supporting Information), which would result in a linear dependence on *H*_TF_. Rather, it is attributed to a field‐induced crossover from the magnetic Kondo condensation to the paramagnetic Fermi liquid phase through the characteristic field *H*_C_.^[^ ^6^ ^]^ Importantly, *H*_C_ is comparable to the field‐induced phase transition field of 1200 G, determined from differential conductance measurements.^[^ ^4^ ^]^ To further explore this field‐induced crossover, we selected three representative fields *H*_TF_ = 50, 100, and 700 G for detailed measurements.

Before proceeding, we recall that the muon Knight shift *K_µ_* is related to the intrinsic spin susceptibility sensed locally by muons. Both *K_µ_* and λ_TF_ in Si:P are nearly temperature‐independent (Figure S3b,c, Supporting Information), indicating an extremely weak Kondo coupling between the local moments and conduction electrons. To discern subtle magnetic correlations,^[^ ^31^ ^]^ we plot ‐*K_µ_T* vs. *T* and λ_TF_/*T* vs. *T* in Figures 4e and S4 (Supporting Information). Here, λ_TF_/*T* is proportional to the imaginary part of the dynamic spin susceptibility λ_TF_/*T*∝χ′′(*q*,ω). Any deviation of ‐*K_µ_T* (λ_TF_/*T*) from *T* ^1^ (*T* ^−1^) behavior signals the development of magnetic correlations beyond the uncorrelated paramagnetic regime.

For *H*_TF_ = 50 G, ‐*K_µ_* (*T*)*T* exhibits a quasi‐linear dependence ‐*K_µ_T* ∼ *T^ξ^* with *ξ =*1.1(1) for *T*_KC_ < *T* < *T* ^*^ = 0.6 K. Deviations from this *T* ^1.1^ dependence become evident above *T* ^*^ = 0.6 K and below *T*_KC,_ *_µ_* = 0.12 K. Meanwhile, λ_TF_(*T*)/*T* increases monotonically down to 0.03 K (Figure S4, Supporting Information). From the log‐log plots in Figures 4e and S4 (Supporting Information), we find λ_TF_/*T* ∼ *T^−ρ^* with *ρ* = 1.08(5) for *T* > *T*_KC_. At higher TFs, *T*_KC_ shifts to lower temperatures, and the anomaly at *T* ^*^ becomes undetectable (Figure 4e). At the same time, the ‐*K_µ_T* ∼ *T* and λ_TF_/*T* ∼ *T ^−^* ^1^ dependences extend over a wider temperature range, supporting the field‐induced suppression of the Kondo condensation, as observed in λ_TF_(*H*_TF_) (Figure 4d). Altogether, the ZF‐ and TF‐*µ*SR results showcase the thermal and field evolution of spin correlations with two characteristic temperatures *T*_KC,_ *_µ_* and *T* ^*^ as well as the critical field *H*_C_. Astonishingly, these features correspond closely to the characteristics identified through point‐contact spectroscopy.^[^ ^6^ ^]^

### 2.3 Thermodynamic Signature of Kondo Condensation

**Figure 5:**
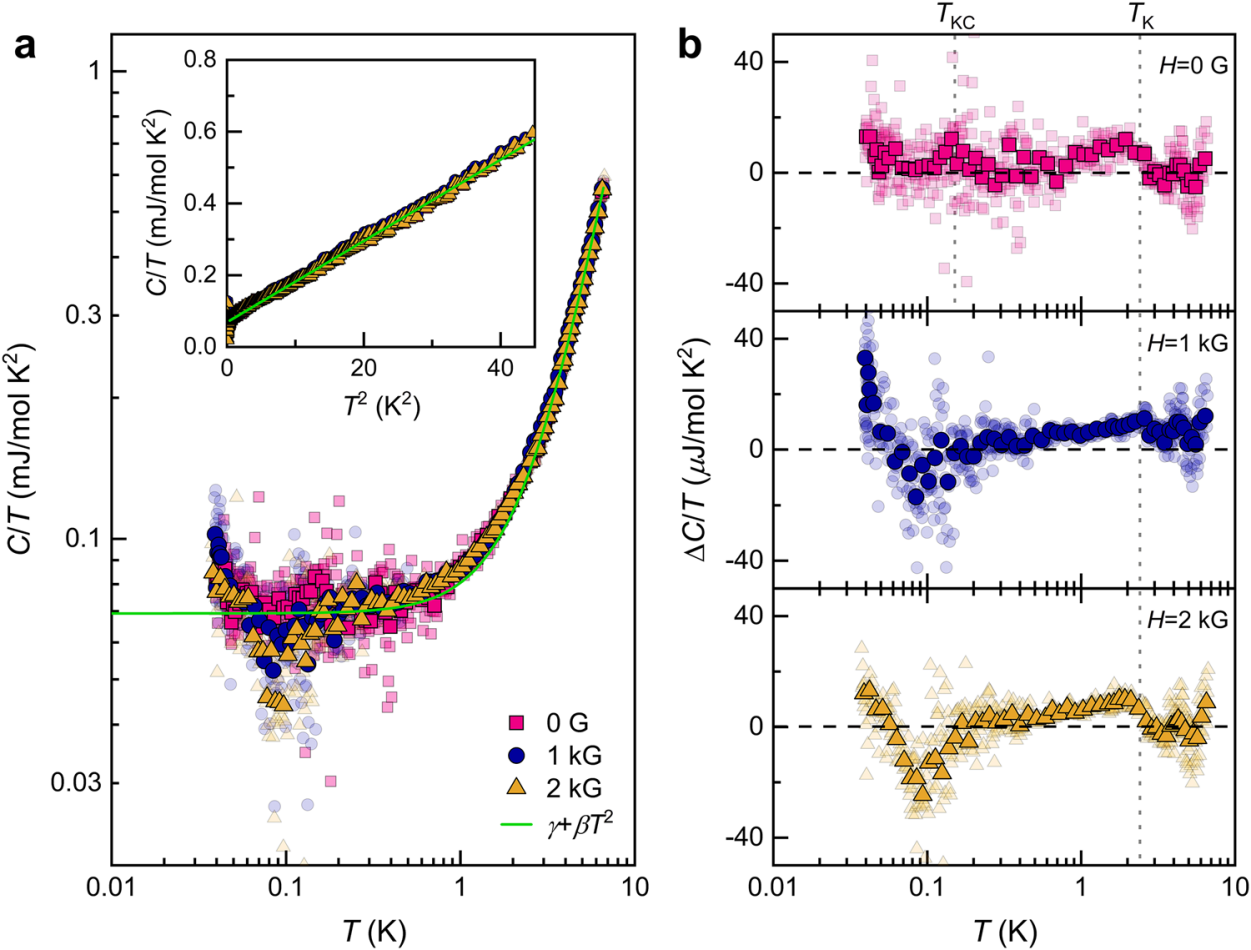
a) Temperature dependence of the specific heat divided by temperature under various magnetic fields. Small blurred symbols represent the raw data, while large clear symbols indicate the median values for every 10 points. The solid line denotes the fits to the data with *C*/*T* = *γ* + *βT* ^2^ above *T* ^*^ = 2.4 K, yielding *γ* = 0.069 mJ/mol K^2^ and *β* = 0.0113 mJ/mol K^4^. The inset shows the *C*/*T* vs. *T* ^2^ plot. b) Temperature dependence of ∆*C*/*T* obtained by subtracting the electronic and lattice contributions from the total specific heat. The horizontal dashed line marks the zero reference.

We further examine thermodynamic evidence of the Kondo condensation and its meltdown by magnetic fields through specific heat measurements on Si:P. As shown in **Figure** **5**a, the total specific heat divided by temperature above *T*_K,_ *_µ_* = 2.4 K (determined by the *µ*SR data) is well described by the Debye‐Sommerfeld equation, *C*/*T* = *γ* + *βT* ^2^, with *γ* = 0.069 mJ/mol∙K^2^ and *β* = 0.0113 mJ/mol K^5^, comparable with earlier reports.^[^ ^36^, ^37^, ^38^ ^]^ Compared to normal metals, the relatively small *γ* value suggests that Si:P is a relatively poor metal, with a low density of states at *E*_F_. After subtracting the electronic and phonon contributions, we obtain the residual specific heat divided by temperature ∆*C*(*T*, *H*)/*T*, as plotted in Figure 5b.

With decreasing temperature, the specific heat difference ∆*C*(*T*, *H* = 0 G)/*T* exhibits a weak hump near *T*_K,_ *_µ_* = 2.4 K, attributed to the formation of a Kondo‐singlet‐like state. Upon further cooling, ∆*C*/*T* shows a discernible peak at around 0.15 K, corresponding to *T*_KC,_ and a subsequent weak upturn below 0.08 K. These anomalies in ∆*C*(*T*, *H* = 0 G)/*T* support the notion that the Kondo singlet formation and the subsequent Kondo condensation are thermodynamically distinct states. In applied magnetic fields, the broad hump of ∆*C*(*T =*2.4 *K*, *H* = 1, and 2 kG)/*T* persists. Meanwhile, the weak peak turns into a dip feature at 0.1 K (attributed to instrumental artifacts as discussed in Figure S7, Supporting Information), followed by a steep increment at low *T* in ∆*C*(*T*, *H* = 1, 2 kG)/*T*. This field‐induced reduction in specific heat at ∼ 0.15 K is linked to the breakdown of the Kondo condensation. Significantly, the suppression of dynamically fluctuating spins results in the closing of the pseudogap state. We further note that the upturn in ∆*C*(*T*, *H*)/*T reflects* a field‐induced modification in the electronic states (Figure S7, Supporting Information).^[^ ^35^ ^]^

## 3 Discussion

By combining optical, thermodynamic, and local resonance probes, we have mapped the full landscape of intriguing Kondo phenomena in highly P‐doped bulk Si, spanning from a Fermi liquid metal through a Kondo‐singlet‐like to a Kondo condensate state. The most salient finding is the presence of critical magnetic fluctuations within the Kondo condensate, with defining characteristics of the Kondo condensation being closely linked between the charge and spin channels.

First, optical and ESR spectroscopy unravels a spin‐fluctuation regime extending over a wide temperature range (20 < *T* < 150–175 K). Given the Kondo temperature (*T*_K_ ∼ 2.4 K) determined from the *µ*SR and specific heat results, it is expected that spin fluctuations would persist up to several tens of Kelvin in a Kondo lattice system. Therefore, the large temperature scale difference between the onset of spin fluctuations and the formation of the Kondo singlet highlights the significant role of disorder. With decreasing temperature below *T*_KC_ ≈ 0.2 K, the overlapping Kondo singlets condense into an incomplete coherent Kondo condensate.

Second, the Kondo condensate state is quite sensitive to external perturbations. Even small applied magnetic fields disrupt the condensate by polarizing the local spins. The crossover fields for the transition from the magnetic Kondo condensation to the paramagnetic Fermi liquid phase are given by *H*_C_ ∼ 988 G from TF‐*µ*SR, and ∼1200 G from point‐contact spectroscopy.^[^ ^6^ ^]^ The similar characteristic fields obtained from distinct methods probing spin and charge excitations advocate the intimate intertwining of spin and charge degrees of freedom in creating the Kondo condensate. This naturally explains why the quenching of collective, correlated spin fluctuations by external fields destabilizes the condensate state.

Third, in the Kondo condensate below *T*_KC_ ≈ 0.2 K, the unscreened local magnetic moments within the overlapping clouds of Kondo singlets are subject to quantum‐critical magnetic fluctuations, as evidenced by the power‐law increase of λ_ZF_(*T*) with no indication of symmetry breaking in the *T* = 0 K limit. The critically fluctuating spins within the Kondo condensate state coexist with a BCS‐like charge gap in the electronic density of states (Ref. [3] and Figure S5, Supporting Information). In this regard, the condensate state of highly P‐doped Si arises from a delicate balance of quantum coherence and quantum‐critical fluctuations. The concurrent critical‐like magnetic fluctuations and charge gap‐defining features of the Kondo condensation state in Si:P‐ closely resemble the pseudogap phases seen in strongly correlated systems such as high‐temperature cuprates, iridates, and unitary Fermi gases.^[^ ^39^, ^40^, ^41^, ^42^, ^43^, ^44^, ^45^, ^46^ ^]^

Qualitatively, the pseudogap observed in Si:P arises from incomplete coherence or fluctuating hybridization among spatially inhomogeneous Kondo clouds. As the Si–P bonding may induce local charge polarization and structural distortions, it may contribute to pseudogap formation. However, the observation that the pseudogap in Si:P is stabilized only at extremely low temperatures and under weak magnetic fields suggests that Si–P bonding is unlikely to be the primary origin. Instead, several microscopic mechanisms should be considered, including translational symmetry breaking, fluctuating spin and charge stripes, string‐net condensation, many‐body pairing, and hidden magnetism. Notably, the Kondo condensate in Si:P entails hallmarks of these pseudogap phases: a loop gas of Kondo singlets featuring intertwined spin and charge fluctuations (see Figure 1c). Within the framework of resonating valence bond theories,^[^ ^41^ ^]^ this pseudogap phenomenon can be envisioned as the charge coherence of overlapping Kondo singlets, fluctuating in a quantum‐critical manner. Alternatively, analogous to unitary Fermi gases,^[^ ^46^ ^]^ pairs of Kondo singlets may undergo Bose‐Einstein‐type condensation in the unitary limit, where strong quantum mechanical scatterings of Kondo singlets prevail over other physical processes. We emphasize that the pseudogap in Si:P remains stable in the zero‐temperature limit, not serving as a precursor phase, and that the onset temperature of the BCS‐like charge gap coincides with the emergence of critical spin fluctuations. As such, our *µ*SR data support the central role of critical magnetic fluctuations as the primary origin of the pseudogap. To pin down its microscopic origins ultimately, future experimental studies should probe hidden symmetry‐breaking orders and associated low‐energy excitations. Additionally, theoretical explanations need to be developed to account for the observed pseudogap behavior. 

## 4 Conclusion

To conclude, we have firmly established highly P‐doped degenerate silicon as a novel platform for investigating the pseudogap phase, driven by critical spin fluctuations of inhomogeneous, overlapping Kondo clouds. The concerted interplay between nonlocal Kondo coherence, long‐range RKKY interactions, and spatial inhomogeneities is a key mechanism underlying this many‐body pairing pseudogap phase. Our findings broaden the scope of pseudogap phenomena, extending their relevance into the realm of doped semiconductors.

## 5 Experimental Section

### Sample Characterization

The doping concentration and crystal orientation of Si:P are ∼2.6 × 10^19^ cm^−3^ and [100], respectively.^[^ ^6^ ^]^ The doping concentration was determined using secondary ion mass spectrometry (SIMS) depth profiling, which confirmed the absence of magnetic impurity atoms. Residual, unscreened localized moments arising from total impurities above ∼2.6 × 10^15^ cm^−3^ are estimated to persist at low temperatures.^[^ ^41^ ^]^ Under these conditions, the mean distance between localized moments is less than 1 *µ*m, comparable to or smaller than the characteristic size of a Kondo cloud.

### Infrared/Optical Spectroscopy

The degenerately P‐doped Si sample was investigated using infrared/optical spectroscopy. Reflectance spectra were measured in a wide spectral range from far‐infrared (FIR) to ultraviolet (UV) (80–40 000 cm^−1^) at various temperatures from 12 to 300 K. To cover this spectral range, a commercial FTIR‐type spectrometer (Vertex 80v; Bruker, Karlsruhe, Germany) was used. A commercial continuous‐flow liquid helium optical cryostat (LT3B Helitran; Advanced Research Systems (ARS), PA, US) was used to cool the sample. An unpolarized beam was employed for the reflectance measurement with an incident angle of ∼10° on the sample. To obtain accurate reflectance spectra, an in situ metallization method was applied by using a coated 200 nm‐thick gold or aluminum film on the sample as a reference reflectance. Gold was used for the far‐ and mid‐IR regions, while aluminum covered the near‐IR, visible, and UV regions. Furthermore, the measured reflectance was corrected by scaling it with the absolute reflection of the gold or aluminum film. Kramers‐Kronig analysis was employed on the measured reflectance spectra to obtain optical constants such as optical conductivity. To perform the Kramers‐Kronig analysis, the reflectance spectrum in a finite spectral range should be extrapolated to zero and infinity. For extrapolation from the lowest data point to zero frequency, the Hagen‐Rubens relation, i.e., $1-R\left(\omega \right)\propto \sqrt{\omega }$, is used. For extrapolation from the highest data point to infinity, up to 10^6^ cm^−1^ *R*(ω)∝ω^−1^ is used, and then above 10^6^ cm^−1^, the free‐electron behavior, i.e., *R*(ω)∝ω^−4^, is assumed.

### Electron Spin Resonance

The ESR measurements were performed with a commercial X‐band ESR system (JEOL JES‐REX3X) at ν = 9.13 GHz. A continuous‐flow helium‐cryostat (ESR900, Oxford Instruments) was used to record ESR signals in the temperature range of *T* = 3.5–280 K. The microwave power dependence of the ESR spectra was taken at *P* = 0.1–200 mW.

For metallic materials, it is known that the metallic surface attenuates the microwave electromagnetic field. Thus, the microwave can penetrate a specimen up to a skin depth *δ*, leading to a so‐called Dysonian lineshape.^[^ ^7^, ^8^ ^]^ The ESR lineshape in a metallic system can be described within the Dyson theory.^[^ ^8^ ^]^ Specifically, the ESR lineshape for the microwave absorption by the resonance of a localized moment is described within the diffusionless limit (1 ≤ *A/B* ≤ 2.6 and *T*_D_/*T*_2_ ≫ 1). Here, *T*_D_ is the average diffusion time of a resonant spin across the skin depth and *T*_2_ is the spin‐spin relaxation time. In the diffusionless limit, the ESR lineshape can be effectively described with the admixture of the absorption *χ*’’ and dispersion *χ*’ of the Lorentzian profiles, given by $\frac{d[\left(1-\alpha \right)\chi^{\prime \prime }+\alpha \chi^{\prime }]}{dH}=\chi_{0}H_{\mathrm{res}}\gamma^{2}T_{2}^{2}\left[\frac{2(1-\alpha )x}{{\left(1+x^{2}\right)}^{2}}+\frac{\alpha (1-x^{2})}{{\left(1+x^{2}\right)}^{2}}\right]$. Here, *x* is defined as *x* = (*H*_res_–*H*)*γT*_2_, *χ*_0_ is the paramagnetic spin susceptibility, *H*_res_ is the resonance field, *H* is the applied field, *γ* is the electronic gyromagnetic ratio, *T*_2_ is the spin‐spin relaxation time, and *α* is the admixture parameter. An effective *g*‐factor was determined by the relation *g* = *hν*/*µ*_B_ *H*_res_. It is noted that the Dysonian asymmetry ratio is given by *A*/*B* = (1 + *α*)/(1 − *α*). In the diffusionless limit, the parameter *α* is constrained to a maximum value of 0.36, which sets a universal upper bound of *A*/*B* = 2.6.^[^ ^7^, ^8^ ^]^ Therefore, an observed ratio of *A*/*B*>2.6 implies *T*_D_/*T*_2_≤1, signaling a crossover to the diffusive Dysonian regime, where coherent spin motion within the skin depth enhances the dispersive component. This criterion provides a practical experimental indicator for distinguishing between diffusionless and diffusive spin dynamics in metallic systems.

### Muon Spin Relaxation/Rotation

Muon spin relaxation/rotation experiments were performed on the FLAME spectrometer at the Paul Scherrer Institute (Villigen, Switzerland). Si:P specimen was sandwiched between a 25 *µ*m Cu foil and wrapped with a Ag foil and mounted on the sample holder. The *µ*SR measurements of Si:P were carried out in the temperature range of *T* = 0.03–10 K and the field range of *H* = 0–3000 G using a Variox He4 cryostat and a KelvinoxJT dilution fridge insert. ZF‐ and TF‐*µ*SR experiments were conducted in the spin‐rotated mode. The initial muon spin is oriented at a 45° angle relative to the muon momentum direction, specifically 45° from the [100] plane of Si:P. The Veto mode was utilized to minimize the background signal. For the TF‐*µ*SR measurements, the transverse field at the sample position, *H*_Ag_, is evaluated using a silver reference for each applied field. All obtained *µ*SR data were analyzed using the MUSRFIT software.^[^ ^47^ ^]^

The ZF‐ and TF‐*µ*SR results were fitted using a single exponential relaxation function: $P_{z}^{\mathrm{ZF}}\left(t\right)=P_{z}\left(0\right)\exp\left(-\lambda_{\mathrm{ZF}}t\right)$ for ZF‐*µ*SR data and $P_{z}^{\mathrm{TF}}\left(t\right)=P_{z}\left(0\right)\exp\left(-\lambda_{\mathrm{TF}}t\right)\cos\left(f_{\mu }t+\phi \right)$ for TF‐*µ*SR data. Here, λ_ZF_ (λ_TF_) is the muon spin relaxation rate in zero‐field (transverse field), *f_µ_* is the frequency of the muon spin precession in the applied transverse field, and *φ* is the initial phase of the muon spin precession. The muon Knight shift was calculated using the expression $K_{\mu }=\left[\frac{H_{\mathrm{Si}:P}-H_{\mathrm{Ag}}}{H_{\mathrm{Ag}}}-\left(\frac{1}{3}-N\right)\chi_{0}\right]\times 100$, where *H*_Si:P_ is the local magnetic field experienced by muons in Si:P, *H*_AG_ is the transverse field at the sample position, *N* is the demagnetization factor (*N* ≈ 1), *χ*_0_ is the bulk susceptibility of Si:P, defined as *χ*_0_ = *M*/*H*_Ag_. Note that no muonium (Mu^0^ = *µ* ^+^ *e* ^−^) signal was found from the *µ*SR data.

### Magnetic and Thermodynamic Characterizations

Magnetic susceptibility and isothermal magnetization data were measured for 16.2 mg of Si:P and 35.3 mg of pure Si (Alfa Aesar, 99.999 %) using a Magnetic Property Measurement System (MPMS, Quantum Design) in the temperature range of *T* = 1.8–400 K and the magnetic field range of *µ*_0_ *H* = 0–7 T. The low‐temperature specific heat was measured using the semi‐adiabatic heat‐pulse method with a self‐made dilution refrigerator. The data in the temperature range of *T* = 2.4 – 7 K, in the absence of a magnetic field, were fitted to a sum of the Debye‐Sommerfeld equation, *C*/*T* = *γ* + *βT* ^2^, to differentiate the electronic, phononic, and other contributions to the total specific heat.

### Statistical Analysis

Optical spectroscopy, ESR, specific‐heat, density‐of‐states spectroscopy, and magnetization data were plotted in their raw form, without any filtering. ZF‐ and LF‐µSR spectra were rebinned into 1500‐time bins using MUSRFIT. TF‐µSR spectra were rebinned into 100‐time bins before applying a fast Fourier transform with a Fourier power of 12. Each µSR dataset comprised ≈3 × 10^7^ positron events collected over ∼1 h per temperature or field point. Statistical analysis and fitting of µSR data were conducted using MUSRFIT, while all other datasets were processed and analyzed in OriginPro (OriginLab Corporation).

## Conflict of Interest

The authors declare no conflict of interest.

## Author Contributions

S.L., S.C., and Y.J. contributed equally to this work. J.H., H.I., and K.C. conceived the project. S.L., W.L, J.K, C.B., T.H., and H.L. performed µSR experiments and analyzed the µSR data. S.L. and Y.O. conducted ESR measurements and analyzed the ESR data. J.K., H.L., and J.H. carried out IR experiments and made data analysis. T.M. and H.I. measured specific heat. E.M. performed magnetization experiments. S.L., S.C., Y.C., J.H., and K.C. wrote the manuscript. All authors took participate in the discussion.

## Supporting information



Supporting Information

## Data Availability

The data that support the findings of this study are available from the corresponding author upon reasonable request.

## References

[1] T. F. Watson , S. G. J. Philips , E. Kawakami , D. R. Ward , P. Scarlino , M. Veldhorst , D. E. Savage , M. G. Lagally , M. Friesen , S. N. Coppersmith , M. A. Eriksson , L. M. K. Vandersypen , Nature 2018, 555, 633. 29443962 10.1038/nature25766

[2] A. Lagendijk , B. van Tiggelen , D. S. Wiersma , Phys. Today 2009, 69, 24.

[3] M. Lee , J. G. Massey , V. L. Nguyen , B. I. Shklovskii , Phys. Rev. B 1999, 60, 1582.

[4] E. Bustarret , C. Marcenat , P. Achatz , J. Kacmarcik , F. Lévy , A. Huxley , L. Ortéga , E. Bourgeois , X. Blase , D. Débarre , J. Boulmer , Nature 2006, 444, 465. 17122852 10.1038/nature05340

[5] H. v. Löhneysen , Ann. Phys. 2011, 523, 599.

[6] H. Im , D. U. Lee , Y. Jo , J. Kim , Y. Chong , W. Song , H. Kim , E. K. Kim , T. Yuk , S.‐J. Sin , S. J. Moon , J. R. Prance , Y. A. Pashkin , J.‐S. Tsai , Nat. Phys. 2023, 19, 676.

[7] M. Lee , Nat. Phys. 2023, 19, 614.

[8] J. Kondo , Prog. Theor. Phys. 1964, 32, 37.

[9] T. F. Rosenbaum , R. F. Milligan , M. A. Paalanen , G. A. Thomas , R. N. Bhatt , W. Lin , Phys. Rev. B 1983, 27, 7509.

[10] M. Lakner , H. v. Löhneysen , A. Langenfeld , P. Wölfle , Phys. Rev. B 1994, 50, 17064. 10.1103/physrevb.50.17064 9976103

[11] P. A. Lee , N. Nagaosa , X.‐G. Wen , Rev. Mod. Phys. 2006, 78, 17.

[12] D. B. Tanner , Optical Effects in Solids, Cambridge University Press, Cambridge, UK 2019.

[13] G. Feher , A. F. Kip , Phys. Rev. 1954, 98, 337.

[14] F. J. Dyson , Phys. Rev. 1955, 98, 349.

[15] R. H. Taylor , Adv. Phys. 1975, 24, 681.

[16] H. J. Spencer , S. Doniach , Phys. Rev. Lett. 1967, 18, 994.

[17] K. Baberschke , E. Tsang , Phys. Rev. Lett. 1980, 45, 1512.

[18] R. J. Elliott , Phys. Rev. 1954, 96, 266.

[19] Y. Yafet , Solid State Physics, 14, Academic Press, Massachusetts, USA 1963.

[20] J. L. Cheng , M. W. Wu , J. Fabian , Phys. Rev. Lett. 2010, 104, 016601. 20366376 10.1103/PhysRevLett.104.016601

[21] J. Park , J.‐J. Zhou , M. Bernardi , Phys. Rev. B 2020, 101, 045202.

[22] D. Chalise , D. G. Cahill , Phys. Rev. Appl. 2023, 20, 064024.

[23] a) J. Sichelschmidt , V. A. Ivanshin , J. Ferstl , C. Geibel , F. Steglich , Phys. Rev. Lett. 2003, 91, 156401; 14611480 10.1103/PhysRevLett.91.156401

[24] xx xx.

[25] J. Korringa , J. Physica 1950, 16, 601.

[26] H. A. K. von Nidda , A. Schütz , M. Heil , B. Elschner , A. Loidl , Appl. Magn. Reson. 1997, 12, 287.

[27] W. Pauli , Nuclear 1927, 41, 81.

[28] L. Landau , Z. für Phys. 1930, 64, 629.

[29] L. C. Hebel , C. P. Slichter , Phys. Rev. 1957, 107, 901.

[30] L. C. Hebel , C. P. Slichter , Phys. Rev. 1959, 113, 1504.

[31] K. Ishida , E. E. MacLaughlin , B.‐L. Young , K. Okamoto , Y. Kawasaki , Y. Kitaoka , G. J. Nieuwenhuys , R. H. Heffner , O. O. Bernal , W. Higemoto , A. Koda , R. Kadono , O. Trovarelli , C. Geibel , F. Steglich , Phys. Rev. B 2003, 68, 184401.

[32] M. Majumder , R. Gupta , H. Luetkens , R. Khasanov , O. Stockert , P. Gegenwart , V. Fritsche , Phys. Rev. B 2022, 105, L180402.

[33] I. Ishant , T. Shiroka , O. Stockert , V. Fritsch , M. Majumder , Phys. Rev. Res. 2024, 6, 023112.

[34] J. E. Sonier , J. H. Brewer , R. F. Kiefl , R. H. Heffner , K. F. Poon , S. L. Stubbs , G. D. Morris , R. I. Miller , W. N. Hardy , R. Liang , D. A. Bonn , J. S. Gardner , C. E. Stronach , N. J. Curro , Phys. Rev. B 2002, 66, 134501.

[35] J. Zhang , Z. Ding , C. Tan , K. Haung , O. O. Bernal , P.‐C. Ho , G. D. Morris , A. D. Hillier , P. K. Biswas , S. P. Cottrell , H. Xiang , X. Yao , D. E. MacLaughlin , L. Shu , Sci. Adv. 2018, 4, aao5235. 10.1126/sciadv.aao5235 PMC5756666 29326982

[36] J. R. Marko , J. P. Harrison , J. D. Quirt , Phys. Rev. B 1974, 10, 2448.

[37] N. Kobayashi , S. Ikehata , S. Kobayashi , W. Sasaki , Solid State Commun. 1977, 24, 67.

[38] H. v. Löhneysen , M. Lakner , Phys. B: Condens. Matter 1990, 165, 285.

[39] T. Timusk , B. Statt , Rep. Prog. Phys. 1999, 62, 61.

[40] M. A. Levin , X.‐G. Wen , Phys. Rev. B 2005, 71, 045110.

[41] P. A. Lee , N. Nagaosa , X.‐G. Wen , Rev. Mod. Phys. 2006, 78, 17.

[42] T. Kondo , R. Khasanov , T. Takeuchi , J. Schmalian , A. Kaminski , Nature 2009, 457, 296. 19148096 10.1038/nature07644

[43] I. Battisti , K. M. Bastiaans , K. M. Bastiaans , V. Fedoseev , A. de la Torre , N. Iliopoulos , A. Tamai , E. C. Hunter , R. S. Perry , J. Zaanen , F. Baumberger , M. P. Allan , Nat. Phys. 2016, 13, 21.

[44] C. S. Chiu , G. Ji , A. Bohrdt , M. Xu , M. Knap , E. Demler , F. Grusdt , M. Greiner , D. Greif , Science 2019, 365, 251. 31320533 10.1126/science.aav3587

[45] M. Frachet , I. Vinograd , R. Zhou , S. Benhabib , S. Wu , H. Mayaffre , S. Krämer , S. K. Ramakrishna , A. P. Reyes , J. Debray , T. Kurosawa , N. Momono , M. Oda , S. Komiya , S. Ono , M. Horio , J. Chang , C. Proust , D. LeBoeuf , M.‐H. Julien , Nat. Phys. 2020, 16, 1064.

[46] X. Li , S. Wang , X. Luo , Y.‐Y. Zhou , K. Xie , H.‐C. Shen , Y.‐Z. Nie , Q. Chen , H. Hu , Y.‐A. Chen , X.‐C. Yao , J.‐W. Pan , Nature 2024, 626, 288. 38326594 10.1038/s41586-023-06964-y

[47] A. Suter , B. M. Wojek , Phys. Proc. 2012, 30, 69.

