# Supplementary material for: Observation of Magnetic Pseudogap Behavior in Phosphorus‐Doped Silicon
Source: Adv Sci (Weinh). 2025 Jul 25;12(39):e02789. doi: 10.1002/advs.202502789 (PMC12533202; doi:10.1002/advs.202502789)
Supplement: Supplementary file 1 — Supporting Information [file ADVS-12-e02789-s001.docx]

Supplementary information

**Observation of magnetic pseudogap behavior in phosphorus-doped silicon**

*Suheon Lee,*^1,10^ *Sangeun Cho,*^2,10^ *Yongcheol Jo*,^3,10^ *Wonjun Lee,*^1^ *Jae Min Kim,*^4^ *Hong Gu Lee,*^4^ *Yugo Oshima,*^5^ *Taku Matsushita,*^6^ *Hiroki Ikegami,*^7^ *Jonas A. Krieger,*^8^ *Christoper Baines,*^8^ *Thomas J. Hicken,*^8^ *Hubertus Luetkens,*^8^ *Eundeok Mun,*^9^ *Jungseek Hwang,*^4*^ *Hyunsik Im,^2,11^ and Kwang-Yong Choi*^4*^

^10^ These authors contribute equally: Suheon Lee, Sangeun Cho, and Yongcheol Jo

^11^ This work is dedicated to the memory of Hyunsik Im, whose vision and initiation of this project made it possible.

^*^ Email to [jungseek@skku.edu](https://mail.worksmobile.com/#/content) and [choisky99@skku.edu](mailto:choisky99@skku.edu)





**Figure S1 a** Microwave power dependence of the ESR spectra at *T* = 40 K. **b** ESR intensity vs. *P_μ_*_W_^1/2^ at 40 K. For metallic systems, it is well established that the ESR intensity is linearly proportional to the square root of the microwave power *I*_ESR_ ~ *P_μ_*_W_^1/2^. With increasing microwave power, the ESR intensity of Si:P manifests the linear behavior and tends to saturate above 40 mW. Upon further increasing *P_μ_*_W_, *I*_ESR_ reaches the maximum at *P_μ_*_W_ = 100 mW and slightly decreases as expected for insulating systems. Such counterintuitive behavior of *I*_ESR_ indicates the coexisting metallic and insulating characters within the skin depth, as evidenced by the metallic Dysonian lineshape and the saturation effects. **c** Microwave power dependence Δ*H*_pp_ (*P_μ_*_W_) of the peak-to-peak ESR linewidth (left) and the spin-spin relaxation time (right) at 40 K. Δ*H*_pp_ (*P_μ_*_W_) exhibits a microwave power-independent behavior up to 10 mW and a subsequent increment for *P_μ_*_W_ > 10 mW. Consequently, *T*_2_ (*P_μ_*_W_) exhibits the opposite behavior to Δ*H*_pp_ (*P_μ_*_W_). The observed anomalous behavior of Δ*H*_pp_ (*P_μ_*_W_) signifies the presence of persistent local fluctuating fields at 40 K developed by the magnetic correlations between the conduction electrons and the localized moments, consistent with the temperature-dependent ESR data. **d** *g*-factor vs. *P_μ_*_W_. **e** Admixture parameter as a function of microwave power. Note that *g* (*P_μ_*_W_) shows a negligible shift with increasing microwave power, while *α* (*P_μ_*_W_) exhibits a monotonic increment. This confirms the diffusionless character of the ESR spectra within the microwave power range *P_μ_*_W_ = 0.1 -200 mW.





**Figure S2** Longitudinal-field dependence of *μ*SR spectra at *T* = 50 mK. The solid curves represent the fittings to the data with a single exponential function. The muon spin relaxation is nearly decoupled with the application of a fairly small longitudinal field of 50 G with a small but finite relaxation rate of 0.0026(9) *µ*s^−1^. Typically, such a low decoupling field is needed for decoupling nuclear moments or weak magnetic order. Assuming the muon depolarization originates from static local fields, λ_ZF_=0.02667 *µ*s^−1^ at 50 mK allows an estimate of the static field distribution width as ∆*H*=0.31 G using the relation ∆*H*~λ_ZF_/*γ*_μ_, where *γ_μ_* is the muon gyromagnetic ratio. In general, the complete decoupling of muon spins from the local static fields in applied longitudinal fields occurs on the order of 10×∆H~3 G. However, the extremely small but residual relaxation at 50 G suggests the presence of dynamic magnetism. In addition, we find that a Gaussian relaxation function is inadequate to describe the LF-*μ*SR spectra. Instead, a simple exponential relaxation function provides a more accurate description of the relaxation behavior. In this light, the rapid decoupling of dynamic magnetism may be due to the weak coupling of local magnetic moments mediated by RKKY interactions in Si:P.





**Figure S3 a** Normalized FFT amplitudes of TF-*μ*SR spectra at the highest and lowest measurement temperatures at *H*_T F_= 50, 100, and 700 G. The solid lines denote the fits to the data. **b** Muon Knight shifts -*K_μ_* as a function of temperature at different transverse fields. **c** Temperature dependence of the muon spin relaxation rates (λ_TF_) at various transverse fields. In order to detect weak magnetic correlations, the muon Knight shift multiplied by temperature -*K*_μ_*T* is plotted in **Figure 4** of the main text, where its implications are discussed.





**Figure S4 a-d** Temperature dependence of the muon spin relaxation rate divided by temperature at different transverse fields of *H*_TF_ = 50, 100, and 700 G. We note that deviations of λ_ZF/TF_/*T* from a *T*^-1^ behavior provide a measure of the development of magnetic correlations. The solid curves represent the power-law behavior of λ_ZF/TF_/*T* ~ *T ^-ρ^*. The dotted and dashed vertical lines indicate the characteristic temperature *T*^*^ and *T*_KC,_*_μ_*, respectively. The shaded regions denote the correlated Kondo singlet phase for *T*_KC,_*_μ_* < *T* < *T*^*^ and the Kondo condensate state below *T*_KC_.





**Figure S5 a** Temperature and field dependence of the d*I*/d*V* curves obtained from density-of-states spectroscopy. A pseudogap of ~ 0.25 meV is observed at temperatures below 100 mK and under magnetic fields below 60 G, indicating that the doping concentration of the sample is sufficiently high to achieve the Kondo condensation. The zero-bias conductance gradually increases with increasing magnetic field. In addition, the pseudogap in the density of states with gap edge peaks disappears as the temperature or magnetic field increases. **b** Phase diagram of Si:P determined by the zero-bias conductance.





**Figure S6 a** Temperature dependence of the magnetic susceptibility in various fields. The inset shows the experimental geometry for the magnetic susceptibility measurements. For all field directions, Si:P manifests a diamagnetic response. The observed diamagnetism becomes stronger when the magnetic field is applied within the sample plane *H*_||_ compared to when applied perpendicular to the plane *H*_⊥_. As the temperature is lowered, *χ*(*T*) of Si:P gradually decreases down to 50 K. Upon further cooling, we find the upturning feature of *χ*(*T*) below 50 K, reflecting paramagnetic contribution by localized moments originating from electrons localized at individual donor sites. It is noteworthy that the susceptibility upturn temperature is comparable with the temperature where the *g*-factor begins to increase (**Figure 3c**). **b** Isothermal magnetization curves of Si:P and Si (Alfa Aesar, 99.999%) at *T*=1.8 K. The inset displays the isothermal magnetization data of Si:P at different temperatures. As the field increases, *M*(*H*) for both field directions shows a typical diamagnetic response with negligible temperature dependence. In addition, the difference in the magnitude of diamagnetic magnetization depending on the magnetic field direction is consistent with the magnetic susceptibility results.





**Figure S7 a** Temperature dependence of the specific heat for Si:P at different fields. **b** Specific heat divided by temperature for Ag in various fields. The dashed lines denote the power-law behavior of the specific heat *C*/*T*~*T*^-^*^η^*. Both Si:P and Ag show a dip of around 0.1 K in applied fields, which is caused by a finite heat leak from the sample holder in the semi-adiabatic pulse method for the specific heat measurements. Nevertheless, we observe the distinct power-law behavior of the low-*T* specific heat, *C*/*T*~*T*^-^*^η^*. The exponent *η* is evaluated as 0.65(5) for Si:P and 0.93(5) for Ag. The change in the exponent value *η* implies the breakdown of Kondo condensates under applied magnetic fields, even considering the influence of experimental artifact around 0.1 K.





**Figure S8** Temperature dependence of the spin-spin relaxation rate on a log-log scale. The solid line indicates the power-law behavior *T*_2_~*T*^-^*^ζ^* with *ζ*=1.44(4). The observed power-law dependence is consistent with the previous study.^[1]^

**Reference**

[1] Darshan Chalise and David G. Cahill, Electron paramagnetic resonance of n-type semiconductors for applications in three-dimensional thermometry, Phys. Rev. Applied **2023**, 20, 064024.
